# Effects of a Home-Based Pulmonary Rehabilitation Program in Patients with Chronic Obstructive Pulmonary Disease in GOLD B Group: A Pilot Study

**DOI:** 10.3390/healthcare9050538

**Published:** 2021-05-04

**Authors:** Rui Vilarinho, Lúcia Serra, Ricardo Coxo, João Carvalho, Cátia Esteves, António Mesquita Montes, Cátia Caneiras

**Affiliations:** 1 Healthcare Department, Nippon Gases Portugal, 2600-242 Lisbon, Portugal; luciaalexandra.alvesserra@externals-nippongases.com (L.S.); ricardo.coxo@nippongases.com (R.C.); prxrrjoaocarvalho@gmail.com (J.C.); catia.milene@nippongases.com (C.E.); catia.caneiras@nippongases.com (C.C.); 2 Department of Physiotherapy and Center for Rehabilitation Research (CIR), School of Health, Polytechnic Institute of Porto, 4200-072 Porto, Portugal; antoniomesquitamontes@gmail.com; 3 Department of Pulmonology, Centro Hospitalar Universitário Lisboa Norte, Lisbon Academic Medical Center, 1649-028 Lisbon, Portugal; 4 Department of Physiotherapy, Santa Maria Health School, 4049-024 Porto, Portugal; 5 Microbiology Research Laboratory on Environmental Health (EnviHealthMicroLab), Faculty of Medicine, Institute of Environmental Health (ISAMB), University of Lisbon, 1649-028 Lisbon, Portugal; 6 Faculty of Medicine, Institute for Preventive Medicine and Public Health, University of Lisbon, 1649-028 Lisbon, Portugal

**Keywords:** chronic respiratory disease, exercise training, self-management, patient-reported outcomes, functional outcomes

## Abstract

Patients with chronic obstructive pulmonary disease (COPD) in the Global Initiative for Chronic Obstructive Lung Disease (GOLD) B group can be included in pulmonary rehabilitation (PR) settings outside the hospitals. This study aimed to explore the feasibility of a home-based pulmonary rehabilitation (HBPR) program and assess its impact on patients with COPD in the GOLD B group. A real-world, pre–post intervention study was conducted with 12 weeks of HBPR (presential home visits and phone calls) using the self-management program Living Well with COPD. The 1-min sit-to-stand test (1MSTS), modified Medical Research Council Questionnaire (mMRC), COPD Assessment Test (CAT), Hospital Anxiety and Depression Scale (HADS), and London Chest Activity of Daily Living (LCADL) were used to assess the impact. Pre–post differences and correlations between changes in outcomes were calculated. In 30 patients (71.6 years, FEV_1_ (%) 52.8), significant improvements (*p* < 0.05) were observed on 1MSTS (Pre 17.2, Post 21.2), mMRC (Pre 2.0, Post 1.0), CAT (Pre 16.3, Post 9.9), HADS (Pre 14.4, Post 9.6), and LCADL (Pre 21.0, Post 15.8), with no adverse events reported. When significant, correlations between changes in outcomes were moderate or strong (0.48 ≤ ρ ≤ 0.66). HBPR can be feasible and safe, and it shows the potential to significantly improve outcomes of patients with COPD in the GOLD B group.

## 1. Introduction

Pulmonary rehabilitation (PR) is a cornerstone of management for people with chronic obstructive pulmonary disease (COPD) presenting well-established benefits [1]. According to the official American Thoracic Society/European Respiratory Society (ATS/ERS) Statement [2], PR can be conducted in a range of settings, where community- and home-based programs present promising results [3,4]. For this reason, a number of factors need to be considered to choose the rehabilitation setting [2], where the assessment of symptoms and risk of exacerbation, according to the ABCD classification system from the Global Initiative for Chronic Obstructive Lung Disease (GOLD) [5], can be included. Patients in the GOLD B group, with more symptoms and less risk of exacerbation, should preferably be included in community- and home-based programs, as opposed to patients with worse classifications and higher risk of exacerbation (GOLD C and D groups) [5], who should be included in hospital programs to ensure safety.

Focusing on home-based programs, their benefits on health-related quality of life (HRQoL) and exercise capacity are comparable to those obtained in hospitals [6,7]. In fact, the development of home-based models is one of the actions undertaken to improve access to and delivery of PR services for suitable patients, due to the needs that hospital programs currently present (costs and accessibility) [8]. Additionally, this setting is, more than ever, an important and necessary solution face to the COVID-19 pandemic, where outpatient programs were advised to suspend their activities [9].

Despite the benefits of home-based pulmonary rehabilitation (HBPR) programs implemented in evidence [6,7], through exercise training and education/self-management protocols in the home environment, most of the studies required the attendance of participants to the hospitals for the assessments before and after programs, especially to assess their benefits on exercise capacity, as a key outcome of PR [2]. The field walking tests, 6-min walk test (6MWT) and incremental/endurance shuttle walk test (ISWT/ESWT) [10], are commonly used to assess it, but their performance in the home environment can present barriers due to lack of space. Therefore, suitable alternatives in order to perform the assessments at patients’ homes are important to analyze the results of HBPR programs. The use of easy functional measurements to assess functional capacity can be recommended. The 1-min sit-to-stand (1MSTS) is an important functional test outcome to be used [11], because it requires minimal resources and space, reflects a common activity in daily living, and has good measurement properties and interpretability [11,12,13].

Therefore, this study aimed to explore the feasibility of an HBPR program by performing both assessment moments and intervention in the home environment, and to assess its impact on functional capacity (1MSTS) and patient-reported outcome measures (PROMs) through symptoms, the impact of the disease, and emotional status in COPD patients in the GOLD B group. Another aim of the present study was to analyze the associations between changes in these outcomes after the HBPR program, especially between the functional capacity outcome (1MSTS) and PROMs. In addition to the expected good feasibility and improvements in all outcomes, we hypothesized that changes in 1MSTS can present significant and moderate to strong correlations with PROMs.

## 2. Materials and Methods

### 2.1. Study Design, Referrals, and Study Criteria

A real-world, pre–post intervention study was conducted in patients with COPD referred by pulmonologists from Portuguese hospitals and clinics. This study was based on the protocol of *reabilitAR* program [14] based on a value-added model of care, with a personalized approach, that contributes to increasing the access of patients with chronic respiratory disease (CRD).

Inclusion criteria were: (i) diagnosis based on GOLD criteria - postbronchodilator forced expiratory volume in 1 second (FEV1)/forced vital capacity (FVC) ratio <70%; (ii) classification of patients in GOLD B group according to the ABCD assessment tool of GOLD criteria [5]; and (iii) electrocardiogram (ECG) record at rest with no significant changes. Exclusion criteria were: (i) presence of any clinical condition that does not allow the participation to the HBPR program, such as, significant cardiovascular (e.g., symptomatic ischemic cardiac disease), neurological (e.g., neuromuscular dystrophy disease) or presence of musculoskeletal disease; (ii) diagnosis of acute exacerbation of COPD according to the GOLD criteria; (iii) signs of cognitive impairment (e.g., dementia); and (iv) severe risk of fall.

This study was conducted according to the best practice orientations of the Directorate-General of Health of Portugal, developed to enhance implementation and use of PR in the national territory [15]. One of its recommendations reflects the choice of PR setting according to the classification system of groups GOLD ABCD, mentioning that patients in the GOLD B group should preferably be included in community- and home-based programs. According to the ABCD assessment tool of GOLD criteria, patients in the GOLD B group present 0 or 1 exacerbation in the previous year (not leading to hospital admission) and symptoms scored by the modified Medical Research Council Questionnaire (mMRC) (≥2) and/or COPD Assessment Test (CAT) (≥10) [5].

Another recommendation of these orientations is the importance of a medical assessment for the inclusion of patients in programs outside the hospitals. For that, all clinical reports of the patients referred to the program were analyzed during the validation process by a pulmonologist (specifically, the ECG and comorbidities), which validated the integration of patients.

Approval for this study was obtained from the ethics committees of the School of Health—Polytechnic Institute of Porto (E0134). Written informed consent was obtained from all participants before any data collection. This study was registered at ClinicalTrials.gov (accessed on 4 May 2021) (registry number NCT04722224).

### 2.2. Sample Size

A sample size estimation with 80% power at 5% significance was calculated, using data from Vaidya and colleagues [11], to detect significant differences in COPD patients’ functional capacity after PR evaluated with the 1-min sit-to-stand test (1MSTS). The pre–post number of repetitions achieved was Pre 19.2 ± 6.1 vs. Post 23.0 ± 7.7 (*p* < 0.001) with an effect size of 0.54, resulting in a total sample size of 29 patients.

### 2.3. Intervention

The program consisted of 12 weeks of HBPR, through presential home visits, for the exercise training and the self-management sessions, and phone calls, including the follow-up of the clinical condition and the progression of exercise training. This program was performed with a total of 14 home visits, with more visits in the first 2 weeks of the program (4 visits). From the third week, one visit was replaced by a phone call, for a total of 10 phone calls. The general objective of this strategic mixture of interventions was to empower the patients to reach a frequency of exercise training between three and five times per week, to grasp their health behavior towards managing their disease conditions [2], and to provide knowledge and strategies face to possible incidences during the program, like detecting alarm/hazard signals to stop exercise training, according to the orientations of the Directorate-General of Health of Portugal [15].

Trained physiotherapists delivered each home visit (duration: 60 min) and phone call.

The HBPR program was based on the self-management educational program Living Well with COPD (LWWCOPD) (available at www.livingwellwithcopd.com, accessed on 04 May 2021) [16].

The exercise training was performed according to guidelines of PR [2] and the module “Integrating an Exercise Program into Your Life” of LWWCOPD. The program included warm-up, endurance, resistance/strength, flexibility, balance training, and a cool-down period. The modified Borg scale was used to measure and monitor the training intensity, especially during endurance training. The endurance training was performed on portable cyclo-ergometers and/or steps/stairs for a target duration of 30 min per session, and the training intensity target was dyspnea or fatigue score of 4 to 6 (modified Borg scale). The resistance/strength training was performed using a variety of tools such as dumbbells, ankle weights, and elastic bands for up to 20 min with exercises of the major upper limbs, lower limbs, and trunk muscle groups. Initial loads equivalent to one that evokes fatigue after 10–12 repetitions, with 1 to 3 sets, were used. The flexibility and balance training components were performed according to the patients’ needs. Flexibility training was done using stretching exercises of two-joint (multijoint) muscles, and balance training was done with stance, transition, and gait exercises. During the program, the progression of the training intensity was also tailored according to the perceived dyspnea and fatigue (modified Borg scale), especially during the phone calls. All the material mentioned above for the exercise training was provided for each patient.

Besides the module on exercise training, each patient received another module from the LWWCOPD entitled “Being Healthy with COPD”, including all educational topics concerning self-management.

### 2.4. Outcome Measures

All assessments were conducted by trained physiotherapists at the patients’ homes.

Socio-demographic information (age, sex), general clinical data (smoking habits, number of exacerbations in the past year, medication, long-term oxygen, noninvasive ventilation, comorbidities), and anthropometric measures (height, weight, body mass index (BMI)) were collected. Additionally, the severity of airflow limitation was classified [5] based on the most recent spirometry from the patients’ clinical notes provided by the referrer doctor.

Study outcomes were assessed before and after the 12 weeks of intervention.

Changes in the number of repetitions of the 1MSTS [11], as a functional capacity outcome, were considered the primary outcome. This test was performed on a normal chair available at the patient’s home with standardized instructions and encouragement. After a test practice, one test was performed, and the number of repetitions was used for analysis. A minimal clinically important difference (MCID) of 3 repetitions was established for patients with stable COPD [11] and used for the analysis of our results.

Changes in dyspnea, the impact of the disease, emotional status, and level of dyspnea during activities of daily living were assessed as secondary outcomes and as patient-reported outcome measures (PROMs) (use of questionnaires and scales to understand the person’s perception of their own health). Dyspnea was assessed with the modified Medical Research Council Questionnaire (mMRC) [17], the impact of the disease was assessed with the COPD Assessment Test (CAT) [18,19], the emotional status was assessed with the Hospital Anxiety and Depression Scale (HADS) [20,21], and the level of dyspnea during activities of daily living was assessed with the London Chest Activity of Daily Living [22]. Minimal clinically important differences (MCIDs) of 1 point for mMRC [23], 2 points for CAT [24], 1.5 points for HADS [25], and 4 points for LCADL [26] were established for patients with stable COPD and used for the analysis of our results.

Symptoms of dyspnea and fatigue, peripheral oxygen saturation (%SpO_2_), heart rate (HR), and blood pressure (BP) at rest were also assessed during the program, in particular, during the monitoring in all home visits. For these measures, the modified Borg scale included in the LWWCOPD program, the pulse oximeter (PalmSAT 2500 Series, Nonin Medical, Minnesota, USA), and the desktop arm blood pressure monitor (GIMA, Gessate, Italy) were used, respectively.

To ensure the safety of the program, especially during the self-managed exercise training sessions without supervision by the physiotherapist, the assessment of balance/fall risk with the Short Form Berg Balance Scale 3-Point (SFBBS-3P) [27] was included before the intervention. The cut-off score of <23 points [27] was used to adapt the scheme of the program by adding more supervised home visits.

The numbers of acute exacerbations in the previous year (before HBPR) and during the program were also registered.

The adverse events were also registered by patients, especially during the self-managed exercise training, or during the home visits and phone calls by the physiotherapist. An adverse event was defined as serious or nonserious. A serious adverse event was defined as an event that leads to hospitalization. On the other hand, a nonserious event was defined as an injury (e.g., musculoskeletal injuries).

### 2.5. Statistical Analysis

Statistical analyses were performed using IBM SPSS Statistics version 27.0 (IBM Corporation, Armonk, NY, USA). The level of significance was set at 0.05. Descriptive statistics were used to describe the sample, and data are presented as mean ± standard deviation or median [percentile 25–75].

Continuous variables were tested for normality with the Shapiro–Wilk test.

Comparisons between pre- and post-intervention outcomes were performed using paired t-test and Wilcoxon signed-rank test. Effect sizes (ES) were calculated for the outcomes and were interpreted as small (≤0.2), medium (≤0.5), or large (≤0.8) [28].

According to the established MCIDs, the number and percentage of patients that improved to a level equal to or above the MCID were calculated.

Correlations between changes in outcomes after HBPR were analyzed using Spearman’s correlations. The strength of correlations was classified according to British Medical Journal guidelines: significant correlation coefficients of 0–0.19 as very weak, 0.2–0.39 as weak, 0.4–0.59 as moderate, 0.6–0.79 as strong, and 0.8–1 as very strong [29].

## 3. Results

**Figure 1:**
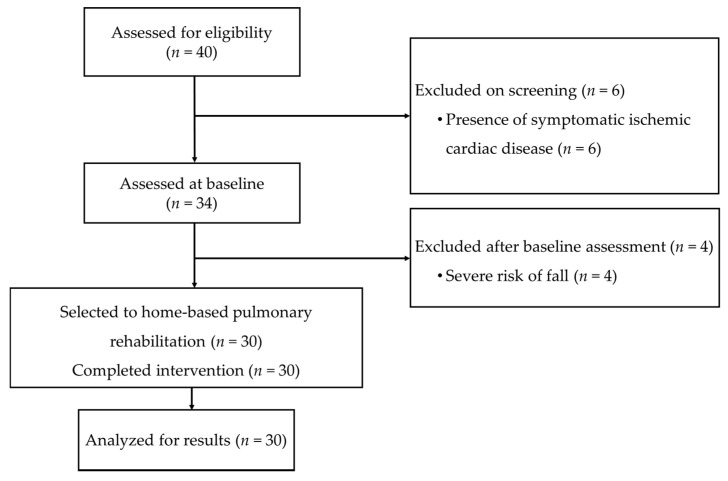
Flow diagram of patients through the study.

Forty patients were referred for possible inclusion in the study. The reasons for the exclusion of six patients were the presence of symptomatic ischemic cardiac disease. In addition, after the baseline assessment, the healthcare professionals responsible for the HBPR program excluded another four patients with severe risk of fall resulting from their high disabilities. Therefore, 30 patients were included in the study (Figure 1).

The characteristics of the 30 patients included in the study are presented in Table 1. The mean body mass index is classified as overweight according to World Health Organization (WHO) criteria [30]. The mean values in vital signs (HR, systolic and diastolic BP) and %SpO2 at rest, obtained at baseline, were considered normal [31].

### 3.1. Impact of the Home-Based Pulmonary Rehabilitation Program

Before HBPR, patients presented preserved balance (SFBBS-3P 25.2 ± 2.9). All patients completed the program, receiving the total number of scheduled home visits and phone calls. After the HBPR program, each patient affirmed the accomplishment of the general objective of reaching a frequency of exercise training between three and five times per week. No adverse events were reported.

In the previous year, patients presented a total of 16 exacerbations; during the HBPR, a total of six exacerbations (four mild, two moderate with antibiotics) were registered.

After the HBPR, significant improvements were found in number of repetitions in 1MSTS (*p* < 0.001). Significant improvements were also found in dyspnea (*p* = 0.002) and fatigue (*p* = 0.048) at rest, both measured by the modified Borg scale, mMRC score (*p* = 0.010), CAT score (*p* < 0.001), total HADS score (*p* = 0.001), HADS-anxiety score (*p* < 0.001), HADS-depression score (*p* = 0.009), and LCADL score (*p* < 0.001) (Table 2).

According to the effect sizes calculated, medium effects were found on dyspnea and fatigue assessed with the modified Borg scale (ES = −0.569; ES = −0.361, respectively), mMRC (ES = −0.468), total HADS score (ES = −0.734), HADS-anxiety score (ES = −0.766), HADS-depression score (ES = −0.531), and LCADL (ES = −0.743). Large effects were found on CAT (ES = −1.020) and 1MSTS (ES = 1.137) (Table 2).

According to the established MCIDs, 15 (50%) patients improved to a level equal to or above the MCID in mMRC score, 22 (77%) patients in total CAT score, 22 (77%) patients in total HADS score, 14 (47%) patients in total LCADL score, and 20 (67%) patients in number of repetitions in 1MSTS.

### 3.2. Correlations between Changes in Outcomes after Home-Based Pulmonary Rehabilitation Program

After HBPR, only one significant correlation was found between the Δ1MSTS and the PROMs, namely with ΔCAT and Δ1MSTS (moderate correlation; ρ: −0.48; *p* = 0.02).

Other significant correlations were found between changes in PROMs classified as moderate or strong. The highest correlation, being classified as strong, was found between ΔCAT and ΔHADS (ρ: 0.66; *p* < 0.001). Another strong correlation was found between ΔmMRC and ΔHADS (ρ: 0.62; *p* = 0.001). Moderate correlations were found between ΔmMRC and ΔCAT (ρ: 0.51; *p* = 0.004) and between ΔmMRC and ΔLCADL (ρ: 0.55; *p* = 0.002). All correlation values are presented in Table 3.

## 4. Discussion

The present study showed that this HBPR program seems to be feasible and showed a positive impact on patients with COPD in the GOLD B group, both in functional capacity outcome (1MSTS) and in PROMs. This study also demonstrates that a home-based model can be safe, as no adverse events were reported. Another important observation was the great adherence of patients in this study, where a high number of scheduled sessions at patients’ homes (14 presential visits and 10 phone calls) [32] and the constant motivation provided by the LWWCOPD program [16] were considered key factors to provide success in adhering to this HBPR program.

To the best of our knowledge, this is one of the first real-world programs where the assessments and the sessions of PR were performed in the patients’ homes [14]. In the scientific evidence, despite the implementation of exercise training and education/self-management protocols in the home environment, most studies required participants to attend the hospitals for the before and after programs of the assessments. This need is explained by the outcomes selected for assessing the programs’ effects, where the exercise test outcomes are the most selected: 6-min walk test (6MWT) [3,4,33,34,35,36,37,38,39,40,41,42,43], incremental shuttle walk test (ISWT) [44,45,46], and endurance shuttle walk test (ESWT) [44,45,46]. Thus, new strategies to assess and analyze the exercise test outcomes, such as the step tests [47], are essential to provide a suitable alternative to the home setting.

Despite the importance of improvements in exercise capacity, according to Meys and colleagues, improvement in exercise test outcomes obtained after PR do not necessarily result in alterations in PROMs in patients with COPD [48]. In fact, functional measurements to assess functional capacity are more recommended, which may better reflect the individual PROM improvements and thus provide a more detailed assessment of the effectiveness of PR programs [48]. This recommendation is especially important in the home setting because it gives an opportunity for direct and thorough analysis of patients’ true ability to fulfill their social roles in real-life situations [49,50]. For this reason, the 1MSTS is an important functional test outcome to be used [49], because it requires minimal resources and space, reflects a common activity in daily living, and has good measurement properties [11,12,13] and interpretability (MCID) [11] for PR. In our results, significant improvements and the highest effect size (ES = −1.137) were demonstrated for 1MSTS, where 20 (67%) patients improved to a level equal to or above the MCID. However, only one significant association was found after PR (Δ1MSTS and ΔCAT (ρ: −0.48; *p* = 0.02)), which did not accurately demonstrate the association of functional capacity and PROMs.

In scientific evidence, the most widely used instruments to assess PROMs in HBPR studies are the Chronic Respiratory Disease Questionnaire (CRQ) [3,4,35,38,40,42,44,46,51] and Saint George’s Respiratory Questionnaire (SGRQ) [4,34,36,37,39,43,44]. However, these instruments were not applied in our program because they require more application time than other questionnaires. The PROMs used in this HBPR program, mMRC, CAT, HADS, and LCADL, are also recommended by the PR guidelines [2], and their administration is simple, clear, and not time-consuming. In the present study, significant improvements and the highest correlations were found in these PROMs after HBPR. Interestingly, the highest correlation found between ΔCAT and ΔHADS (ρ: 0.66; *p* < 0.001) can indicate that the influence of HBPR on the impact of symptoms (assessed by CAT) is strongly associated with the improvements in the emotional status of patients (assessed by HADS). This result is important since CAT is a multidimensional disease-specific PROM, which assesses multiple symptoms in COPD, unlike the mMRC, with the highest number of significant correlations with other PROMs in this study, but it is considered a unidimensional questionnaire to quantify only dyspnea [2]. Furthermore, the application of HADS has been a target of interest in evidence, especially on HBPR with promising results [3,46,52]. One of the reasons is the ability of this questionnaire to identify barriers influencing the PR self-efficacy in the home environment and factors that can help create important strategies for the self-management intervention [2].

A strength of this HBPR program is the potential to induce a prolonged self-management routine, especially exercise training, facilitated by the home environment and the LWWCOPD program. According to Lahham and colleagues, in a qualitative study reporting the perspectives of COPD patients who underwent HBPR, many participants reported they had established a routine that they were committed to continuing after the program [53]. Therefore, futures studies are important to assess the follow-up of people after this HBPR program to confirm one of the recommendations of the ATS/ERS recommendations: the long-term adherence to health-enhancing behaviors, as a key goal of PR for patients with COPD [2]. Another important topic is the study of new strategies to enhance the access to HBPR in advanced groups of GOLD ABCD classification, specifically GOLD C and D, because despite the importance of safety during rehabilitation, these severe patients face more barriers, including the absence of national programs in nonurban geographic areas. Scientific evidence already suggests that HBPR can be safe and have improvements on outcomes in these advanced patients [37,51].

As a limitation of our study, the use of a pre–post intervention study does not allow definitive causal inferences. For this purpose, future studies should consider the use of a control group. However, evidence already indicates improvements and clinical outcomes equivalent to those of the traditional hospital-based programs, in addition to higher completion rates and high acceptability of the home-based intervention [3,4]. Another limitation is the reduced number of functional test outcomes used in this study. Further studies should include more functional tests, assess their responsiveness to PR, and analyze their association with PROMs in order to conduct a more detailed assessment of the effectiveness of PR programs, especially those of the home-based models.

## 5. Conclusions

This HBPR can be feasible, and it shows potential to significantly improve functional capacity and PROMs in patients with COPD in the GOLD B group. Significant associations were observed between PROMs; however, only one significant correlation was found between the functional capacity outcome (1MSTS) and PROMs, namely with CAT. This study also demonstrates that this home-based model is safe, as no adverse events were reported during the program. Future research should focus on the study of the follow-up of people after this HBPR program. Moreover, it is important to include other GOLD groups (C and D groups) and add more functional test outcomes to determine their responsiveness in PR.

## Figures and Tables

**Table 1:** Participant characteristics.

Characteristics	*n* = 30
Age, years	71.6 ± 9.4
Sex, male/female (%)	12/18 (40.0/60.0)
BMI, kg/m^2^	26.4 ± 5.3
GOLD stages, *n* (%)	
I	2 (6.6)
II	11 (36.7)
III	16 (53.4)
IV	1 (3.3)
FEV_1_, %predicted	52.8 ± 18.3
FEV_1_/FVC (%)	50.6 ± 11.9
Noninvasive ventilation, *n* (%)	9 (30.0)
Long-term oxygen therapy, *n* (%)	9 (30.0)
Comorbid illness, *n* (%)	
Coronary artery disease	5 (17)
Arrhythmia	6 (20)
Hypertension	11 (37)
Diabetes	6 (20)
Musculoskeletal	11 (37)
Medication, *n* (%)	
SABA	5 (17)
LABA	4 (13)
SAMA	4 (13)
LAMA	9 (30)
LABA + LAMA	11 (37)
LABA + ICS	11 (37)
LABA + LAMA + ICS	2 (7)
ICS	11 (37)
Xanthines	11 (37)
Heart rate at rest (bpm)	76.7 ± 12.1
Systolic BP at rest (mmHg)	133.6 ± 15.7
Diastolic BP at rest (mmHg)	73.3 ± 11.3
SpO_2_ at rest (%)	94.1 ± 2.8

Data are expressed as mean ± standard deviation, unless otherwise stated. BMI, body mass index; GOLD, Global Initiative for Chronic Obstructive Lung Disease; FEV1, forced expiratory volume in 1 s; FVC, forced vital capacity; SABA, short-acting β2-agonists; LABA, long-acting β2-agonists; SAMA, short-acting muscarinic antagonists; LAMA, long-acting muscarinic antagonists; ICS, inhaled corticosteroids; BP, blood pressure; SpO2, peripheral oxygen saturation.

**Table 2:** Outcome values before and after home-based pulmonary rehabilitation.

Variables (*n* = 30)	Pre-HBPR	Post-HBPR	*p* Value	ES
1MSTS (repetitions)	17.2 ± 4.4	21.2 ± 5.3	<0.001	1.137
Dyspnea (mBorg)	1.0 [0.0; 3.0]	0.0 [0.0; 0.3]	0.002	−0.569
Fatigue (mBorg)	0.0 [0.0; 1.3]	0.0 [0.0; 0.0]	0.048	−0.361
mMRC (score)	2.0 [1.0; 2.5]	1.0 [1.0; 2.0]	0.010	−0.468
CAT (score)	16.3 ± 4.9	9.9 ± 5.2	<0.001	−1.020
HADS (score)	14.4 ± 5.8	9.6 ± 5.8	0.001	−0.734
HADS A (score)	7.8 ± 4.2	5.1 ± 3.4	<0.001	−0.766
HADS D (score)	6.6 ± 2.8	4.6 ± 3.1	0.009	−0.531
LCADL (score)	21.0 ± 7.4	15.8± 3.3	<0.001	−0.743

Data are expressed as mean ± standard deviation or median [percentile 25–75]. HBPR, home-based pulmonary rehabilitation; ES, effect size; mBorg, modified Borg scale; mMRC, modified Medical Research Council; CAT, COPD Assessment Test; HADS, The Hospital Anxiety and Depression scale; A, anxiety; D, depression; LCADL, London Chest Activity of Daily Living; 1MSTS, 1-min sit-to-stand.

**Table 3:** Correlations between changes in outcomes after home-based pulmonary rehabilitation.

Outcomes	∆1MSTS	∆mMRC	∆CAT	∆HADS	∆LCADL
∆1MSTS	---	NS	−0.48 *	NS	NS
∆mMRC	---	---	0.51 **	0.62 **	0.55 **
∆CAT	---	---	---	0.66 ***	NS
∆HADS	---	---	---	---	NS
∆LCADL	---	---	---	---	---

Correlations are reported as Spearman’s ρ; * *p* < 0.05, ** *p* < 0.01, *** *p* < 0.001; mMRC, modified Medical Research Council; CAT, COPD Assessment Test; HADS, Hospital Anxiety and Depression Scale; LCADL, London Chest Activity of Daily Living; 1MSTS, 1-min sit-to-stand; NS, nonsignificant.

## Data Availability

Data sharing not applicable.
